# Transcriptomic Analysis Reveals the Molecular Adaptation of Three Major Secondary Metabolic Pathways to Multiple Macronutrient Starvation in Tea (*Camellia sinensis*)

**DOI:** 10.3390/genes11030241

**Published:** 2020-02-25

**Authors:** Hui Su, Xueying Zhang, Yuqing He, Linying Li, Yuefei Wang, Gaojie Hong, Ping Xu

**Affiliations:** 1 Department of Tea Science, Zhejiang University, Hangzhou 310058, China; su10928@163.com (H.S.); zdcy@zju.edu.cn (Y.W.); 2 State Key Laboratory for Quality and Safety of Agro-products, Institute of Virology and Biotechnology, Zhejiang Academy of Agricultural Sciences, Hangzhou 310021, China; zhangxuey89@163.com (X.Z.); yqhe_123@126.com (Y.H.); anran6297@163.com (L.L.)

**Keywords:** *Camellia sinensis*, transcriptomics, macronutrient starvation, secondary metabolite, transcription factors

## Abstract

Tea (*Camellia sinensis* (L.) O. Kuntze) is a widely consumed beverage. Lack of macronutrients is a major cause of tea yield and quality losses. Though the effects of macronutrient starvation on tea metabolism have been studied, little is known about their molecular mechanisms. Hence, we investigated changes in the gene expression of tea plants under nitrogen (N), phosphate (P), and potassium (K) deficient conditions by RNA-sequencing. A total of 9103 differentially expressed genes (DEG) were identified. Function enrichment analysis showed that many biological processes and pathways were common to N, P, and K starvation. In particular, cis-element analysis of promoter of genes uncovered that members of the WRKY, MYB, bHLH, NF-Y, NAC, Trihelix, and GATA families were more likely to regulate genes involved in catechins, l-theanine, and caffeine biosynthetic pathways. Our results provide a comprehensive insight into the mechanisms of responses to N, P, and K starvation, and a global basis for the improvement of tea quality and molecular breeding.

## 1. Introduction

Nitrogen (N), phosphate (P), and potassium (K) are well established as primary nutrients for plants and have crucial roles in numerous biological processes [1,2]. To cope with adversity and thrive in different conditions, plants have evolved a myriad of refined but intricate systems, and can adapt to environmental stress through reprogramming their physiological and biochemical processes [3,4].

Over the last decades, remarkable progress has been made in our understanding of N, P, and K signaling and homeostasis mechanisms [5,6,7]. N is not only a major component of a variety of compounds, but also serves as a signaling molecule [8]. The nitrate transporter chlorina 1/nitrate transporter 1.1 (CHL1/NRT1.1), which can detect internal N/NO_3−_ availability, is a key player in the N signaling pathway [9]. Phosphate starvation response 1 (PHR1) in *Arabidopsis*, a member of the MYB-CC family, and its homologs in other plants, acts as master regulator of Pi homeostasis, and miR399, PHO2, SPX-containing proteins also modulate a series of downstream phosphate starvation-induced genes [10]. When challenged by K shortage, plant expression of high-affinity K transporter HAK5 is increased, and AKT1, an influx K channel, is phosphorylated by CIPK23 (Calcineurin B-like interacting protein kinase23), which is also responsible for responses to K deprivation by enhancing AKT1 uptake activity [11].

Recently, researchers have focused on the molecular links among nutrient signaling pathways that sustain the nutrition balance in plants. In *Arabidopsis*, the effect of N on P starvation responses (PSR), by a combination of local and long-distance N signaling pathways, was identified. It has been demonstrated that CHL1/NRT1 activity is dependent on the accumulation and turnover of N by the transcription factor PSR1/PHR1, and that phosphate 2 (PHO2) functions as an integrator of N availability into the PSR [12]. It has also been recently revealed that the N and P signaling networks are integrated through the Nitrate-NRT1.1B-SPX4 cascade. Nitrate perception strengthens the interaction between nitrate sensor NRT1.1B and a phosphate signaling repressor SPX4, and further accelerates the ubiquitination and degradation of SPX4 by recruiting NRT1.1B interacting protein 1 (NBIP1), an E3 ubiquitin ligase [13]. In addition, the association between N and K has also been expounded. N deficiency induces leaf senescence, and a recent study demonstrated that NRT1.5 is essential in perceiving signals derived from nitrate starvation, hence facilitating foliar K accumulation to suppress nitrate starvation-induced leaf senescence [14]. These lines of evidence strongly imply the existence of molecular crosstalk between multiple macronutrients.

Tea is an important cash crop with various components that contribute to its health benefits [15]. Like other plants, tea can suffer from nutrient stress, which subsequently leads to changes in metabolite content, ultimately affecting its quality and medical value [16,17]. At present, there is little information about the secondary metabolite biosynthesis, or the genes and pathways modulated by N, P, and K starvation. For several decades now, RNA-sequencing has been a powerful tool for analyzing potential actors under any given condition, including macronutrient limitation [18,19,20,21,22]. Here, we reported the effects of macronutrient starvation on tea through RNA-seq. By comparing the transcriptional level of key genes from three major secondary metabolite biosynthesis pathways, we found that N, P, and K nutrient-starvation led to different expression patterns. Subsequent transcriptome analysis of these data revealed a total of 9103 differentially expressed genes (DEGs), of which, 115 genes with 26 up-regulation and 89 down-regulation. Interestingly, we also found seven Kyoto Encyclopedia of Genes and Genomes (KEGG) pathways shared by these macronutrients, indicating potential crosstalk among multiple macronutrients. Quantitative reverse transcription polymerase chain reaction (qRT-PCR) and metabolite determination were conducted to verify the responses to nutrient stress. The comparison of transcriptome responsiveness to different nutrient starvation conditions gives a full picture of potential transcriptional regulatory mechanisms responsible for nutrition starvation responses, which is meaningful for tea quality improvement and molecular breeding.

## 2. Materials and Methods

### 2.1. Plant Materials and Stress Treatments

One-year old Longjing 43 (*Camellia sinensis*) seedlings were selected for nutrient starvation treatment. The tea plants were grown hydroponically in distilled water for 7 d and then exposed stepwise to 1/8, 1/4, 1/2, and then full-strength nutrient solution for two months. The nutrient solution contained: (NH_4_)_2_SO_4_: 0.713; KNO_3_: 0.73; KH_2_PO_4_: 0.1; K_2_SO_4_: 0.46; CaCl_2_: 0.5; MgSO_4_: 0.41 mmol·L^−1^; Fe-EDTA: 0.032; H3BO3: 0.046; CuSO_4_: 0.002; MnSO_4_: 0.09; Na_2_MoO_4_: 0.0026; ZnSO_4_·7H_2_O: 0.0091; Al_2_(SO_4_)_3_: 0.04 μmol·L^−1^(Table S1). The nutrient solutions were changed weekly, and the pH value was adjusted to approximately 5.0. The plants were grown with normal nutrient solutions in chambers under a day/night ratio of 14/10 and 40% relative humidity at a temperature of approximately 25 °C. After two months of complete nutrition solution culture, the nutrient starvation treatments were performed as follows: NH_4_^+^, NO_3_^−^_,_ H_2_PO_4_^−^, and K were replaced by Na^+^, Cl^−^, Cl^−^, and Na^+^, respectively for 30 d for N, P, and K starvation treatment, which was revealed by the relative expression of responsive genes (Table S2). Thirty hydroponic tea seedlings were used in each treatment and control group, five tea seedling leaves were randomly selected as biological replicates, three biological replicates were conducted for each group. All the harvested samples were immediately frozen in liquid nitrogen and stored at −80 °C for further use.

### 2.2. Library Construction and Sequencing

Total RNA was isolated using the Trizol Reagent (Invitrogen Life Technologies, Carlsbad, CA, USA), after which the concentration, quality, and integrity were determined using a NanoDrop spectrophotometer (Thermo Scientific, Waltham, MA, USA) and Agilent 2100 Bioanalyzer (Agilent Technologies, Wilmington, DE, USA). Twelve independent leaf complementary DNA (cDNA) libraries which stand for four different experimental conditions with three biological repetitions were constructed using the Illumina Gene Expression Sample Preparation Kit according to the manufacturer’s instructions, and 125 paired end reads were sequenced at the Norwegian Sequencing Centre. All the sequenced data is available at National Center for Biotechnology Information (NCBI) under the bioproject accession number PRJNA602126.

### 2.3. Quantitative Reverse Transcription Polymerase Chain Reaction (qRT-PCR) Analysis

Quantitative RT-PCR analysis for validation was performed by SYBR Green monitored qPCR with the following reaction conditions: 95 °C for 30 s, 40 cycles of 95 °C for 5 s, 60°C for 15 s, and 72 °C for 30 s. Three biological replications were included. Relative expression levels were calculated as the ratio of candidate gene expression to housekeeping gene *CsGADPH* (Glyma.17G186600) expression as described previously. The expression levels of 15 DEGs were calculated according to Livak and Schmittgen [23].

### 2.4. Extraction and Determination of Catechins, l-theanine, and Caffeine in Tea

Catechins and caffeine were extracted and detected by a modified method described by Li et al. [24]. Briefly, 0.1 g of freeze-dried tea leaves was ground in liquid nitrogen with a mortar and pestle, and extracted with 5 mL of 80% methanol in an ultrasonic sonicator for 10 min at 4 °C. After centrifugation at 3000 rpm for 10 min at 4 °C, the residues were re-extracted twice as described above. The supernatants were combined and diluted with 80% methanol to a volume of 10 mL. The obtained supernatants were filtered through a 0.22 μm organic membrane before high-performance liquid chromatography (HPLC) analysis. The catechins and caffeine were measured using the Agilent 1100 HPLC system equipped with a quaternary pump and an ultraviolet (UV)-visible detector. Agilent TC-C_18_ columns (Phenomenex 250 mm × 4.6 mm, 5 μm) were used and the samples were eluted at 40 °C at a flow rate of 1 mL/min. The detection wavelength was set to 280 nm. The mobile phase A consisted of 3% (v/v) acetonitrile, 0.5% (v/v) acetic acid, and 96.5% water and the B of 30% (v/v) acetonitrile, 0.5% (v/v) acetic acid, and 69.5% water; the gradient elution was as follows: B 20–80% at 10 min. Then, 10 μL of the filtrate was injected into the HPLC system for analysis.

Freeze-dried tea leaves weighing 0.15~g were ground in liquid nitrogen with a mortar and pestle, and 25 mL deionized water was added into the sample, which was incubated for 40 min in a water bath at 100 °C. After low centrifugation for 10 min, the residues were re-extracted once as described above. The supernatants were combined and diluted with water to a volume of 25 mL. The obtained samples were then treated according to the OPA method, which is as follows: 10 μL of sample was added to 1 mL BA (boric acid) buffer solution, 200 μL OPA solution (0.08 g OPA (O-phthalaldehyde), 125 μL β-mercaptopropanol, 7 mL BA and 1 mL acetonitrile ), and 800 μL water, and then filtered through a 0.45 μm water membrane for HPLC-FLD analysis. The mobile phase A consisted of 40 mM/L disodium hydrogen phosphate and the counterpart B of 450 mL methanol, 450 mL acetonitrile, and 100 mL water. l-theanine was detected using a LC-20AD HPLC system equipped with a binary pump and FLD detector. A Zorbax eclipse-AAA column (Phenomenex 150 mm × 4.6 mm, 3.5 μm) was used at a flow rate of 1.5 mL/min. The gradient elution was as follows: 0% (v/v) to 60% at 27 min, to 100% at 34.50 min. Then, 10 μL of the filtrate was injected into the HPLC system for analysis. 

## 3. Results

### 3.1. Transcriptome Analysis of Tea Plant Responses to Macronutrient Deficiency

Since N, P, and K play critical roles in tea plants, we performed transcriptome sequence analysis to obtain a genome-wide view of molecular responsiveness in the 12 libraries. Approximately 6.95 Gb clean reads had Q30 values not less than 90.12%. The average number of reads that mapped to tea genome (http://tpia.teaplant.org/download.html) ranged from 89.51% to 90.05%. A total of 39,945 genes were identified, of which, 10,873 were novel genes with 8159 having functional annotations (Tables S3–S4). 

### 3.2. Identification of Differentially Expressed Genes under Nitrogen (N), Phosphate (P), and Potassium (K) Starvation Conditions

**Figure 1:**
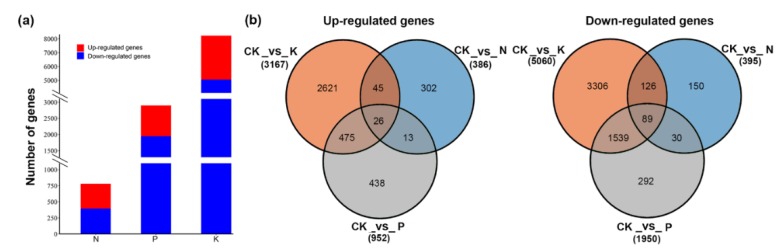
The distribution of differentially expressed genes under nitrogen (N), phosphate (P), and potassium (K) starvation. (**a**) The number of differentially expressed genes under N, P, and K starvation. (**b**) The number of up-regulated genes specific to and overlapping in N, P, and K starvation. (**c**) The number of down-regulated gene specific to and overlapping in N, P, and K starvation.

**Figure 2:**
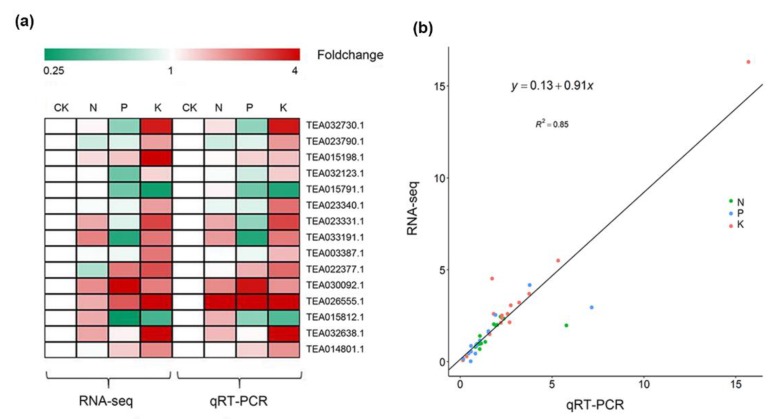
Quantitative real-time polymerase chain reaction (qRT-PCR) validation of 15 differentially expressed genes (DEGs). (**a**) Heatmap of transcript levels of 15 DEGs and the corresponding expression of RNA-seq. (**b**) Comparison of the relative expression obtained from RNA-Seq data and qRT-PCR. The RNA-seq fold-change of the relative expression (*y*-axis) has been plotted against the relative expression of qRT-PCR analysis (*x*-axis).

To decipher the genomic reprogramming framework under corresponding macronutrient stresses, differentially expressed genes (DEGs) were examined with the criteria log2 fold-change |(Log2FC|) ≥ 1 and false discovery rate (FDR) ≤ 0.01. A total of 9103 genes were found to differentially express under N, P, and K starvation stress. Of these, the highest number of DEGs was in the K (8227) group, followed by P (2902) and N (781) compared with the control sample (Tables S5–S7). Following N starvation, 386 identified genes were significantly up-regulated, which was almost equal to the number of markedly down-regulated genes (395). The number of down-regulated genes was larger than that of up-regulated genes in response to P and K deficiency. P starvation induced the up-regulation of 952 DEGs but the down-regulation of 1950 DEGs (Figure 1). Notably, K deprivation brought about sharp changes in transcript levels, with 3167 and 5060 genes significantly induced and repressed, respectively (Figure 1a,b). The different treatment groups had both exclusive and overlapping gene DEGs. Except for the distinctive part from each other, 158 (39 up-regulated, 119 down-regulated), 286 (71 up-regulated, 215 down-regulated), and 2129 (501 up-regulated, 1628 down-regulated) genes with the same expression pattern were common to N and P, N and K, and P and K deprived plants, respectively. More importantly, 115 genes with 26 up-regulation and 89 down-regulation were common to all treatments. In addition, comparison of expression patterns revealed that there were more or less overlaps with each other (Figure 1b). These results imply the possibility of molecular connections among three macronutrient starvation-induced responses. To confirm the validity of RNA-seq data, 15 DEGs involved in metabolite synthesis, transport, or as transcription factors were selected for qRT-PCR analysis. The expression trends of the qRT-PCR data were in accordance with the RNA-seq data (Figure 2). There was a significant correlation between the fold change in gene expression observed through FPKM (Fragments Per Kilobase of transcript per Million fragments mapped) values and qRT-PCR (Pearson correlation coefficient R^2^ = 0.85, *p*-value < 0.0001), indicating the reliability of our RNA-seq data. 

### 3.3. Function Enrichment Analysis of Common Differentially Expressed Genes (DEG) 

**Figure 3:**
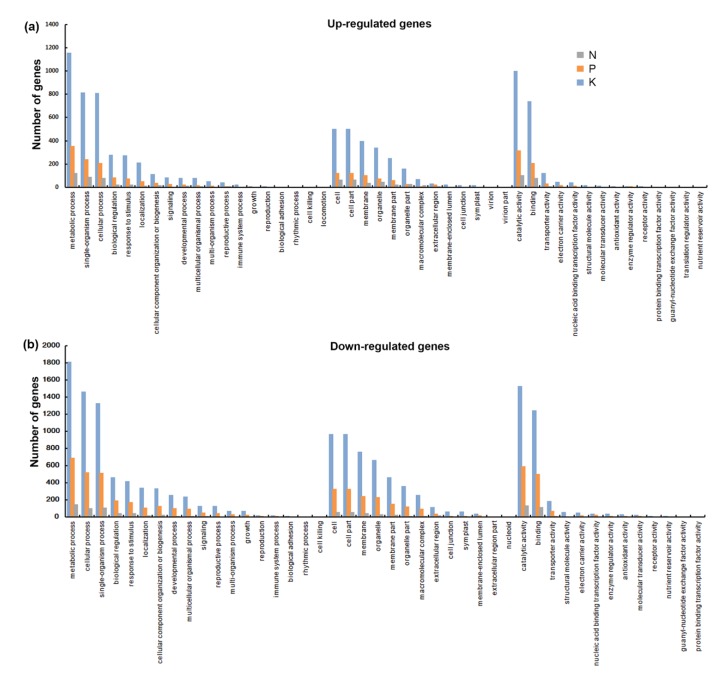
Gene Ontology (GO) annotation of differentially expressed genes under N, P, and K starvation. (**a**) GO classification of up-regulated genes under N, P, and K starvation. (**b**) GO classification of down-regulated genes under N, P, and K starvation. The *x*-axis represents the GO classification and the *y*-axis represents the number of differentially expressed genes mapped to the GO database.

**Figure 4:**
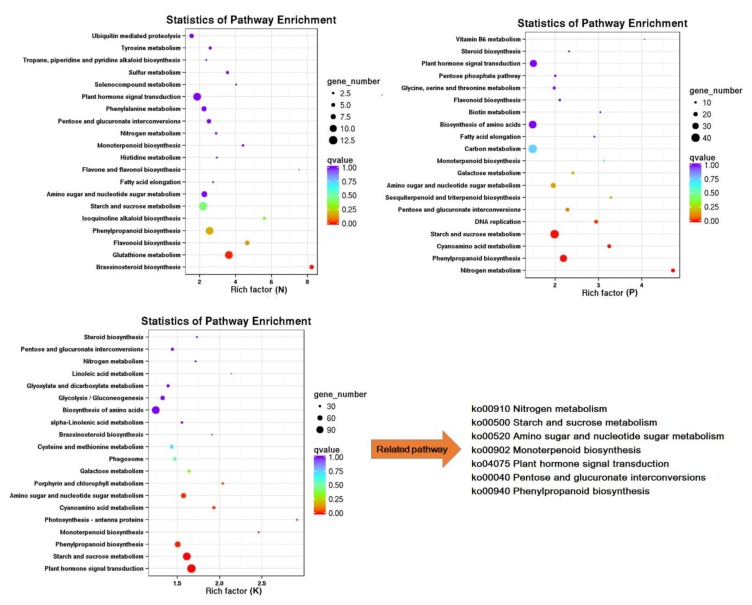
Kyoto Encyclopedia of Genes and Genomes (KEGG) pathway enrichment of differential expressed genes under N, P, and K starvation stress. The top 20 significantly enriched KEGG terms were selected. Each circle represents a KEGG pathway, the *x*-axis represents the enrichment factor, and the *y*-axis the name of KEGG pathway. The color of the circle refers to the *q*-value, and the size of the circle represents the number of genes mapped to the pathway.

In order to gain an insight into the biological functions of these responsive genes, we carried out annotation enrichment using Gene Ontology (GO) and Kyoto Encyclopedia of Genes and Genomes (KEGG) databases. Gene Ontology term analysis (Q < 0.05) categorized the DEGs into biological process, molecular function, and cellular component categories. As shown in Figure 3, though several processes were exclusive to particular treatments, for example, sulfate assimilation was only detected in N starvation but not in others, most over-presented biological processes in the N starvation group were also found in the P and K starvation groups, including DNA integration, protein ubiquitination, and peptidyl-tyrosine phosphorylation. Similarly, most GO terms in the cellular component and molecular function categories involved were common to all macronutrient starvation groups, suggesting that there is crosstalk through manipulation of common biological functions under macronutrients stress. This was further verified by KEGG analysis. Data from the top 20 KEGG enrichment pathways showed that pathways in the N starvation group, such as flavonoid biosynthesis, fatty acid elongation, and brassinosteroid biosynthesis were also influenced by P starvation and K starvation. Similarly, K deficiency affected cyanoamino acid metabolism, galactose metabolism, biosynthesis of amino acids, and steroid biosynthesis, which were also involved in the response to P deficiency (Figure 4). Additionally, seven shared pathways among each of the group were noteworthy, they were: nitrogen metabolism, starch and sucrose metabolism, amino sugar and nucleotide sugar metabolism, monoterpenoid biosynthesis, plant hormone signal transduction, pentose and glucuronate interconversions, and phenylpropanoid biosynthesis. Overall, function enrichment analysis strongly indicated the existence of molecular networks in the responses to these macronutrient stresses.

### 3.4. The Effect of N, P, and K Starvation on Three Major Secondary Metabolite Biosynthesis Pathways in Tea

**Figure 5:**
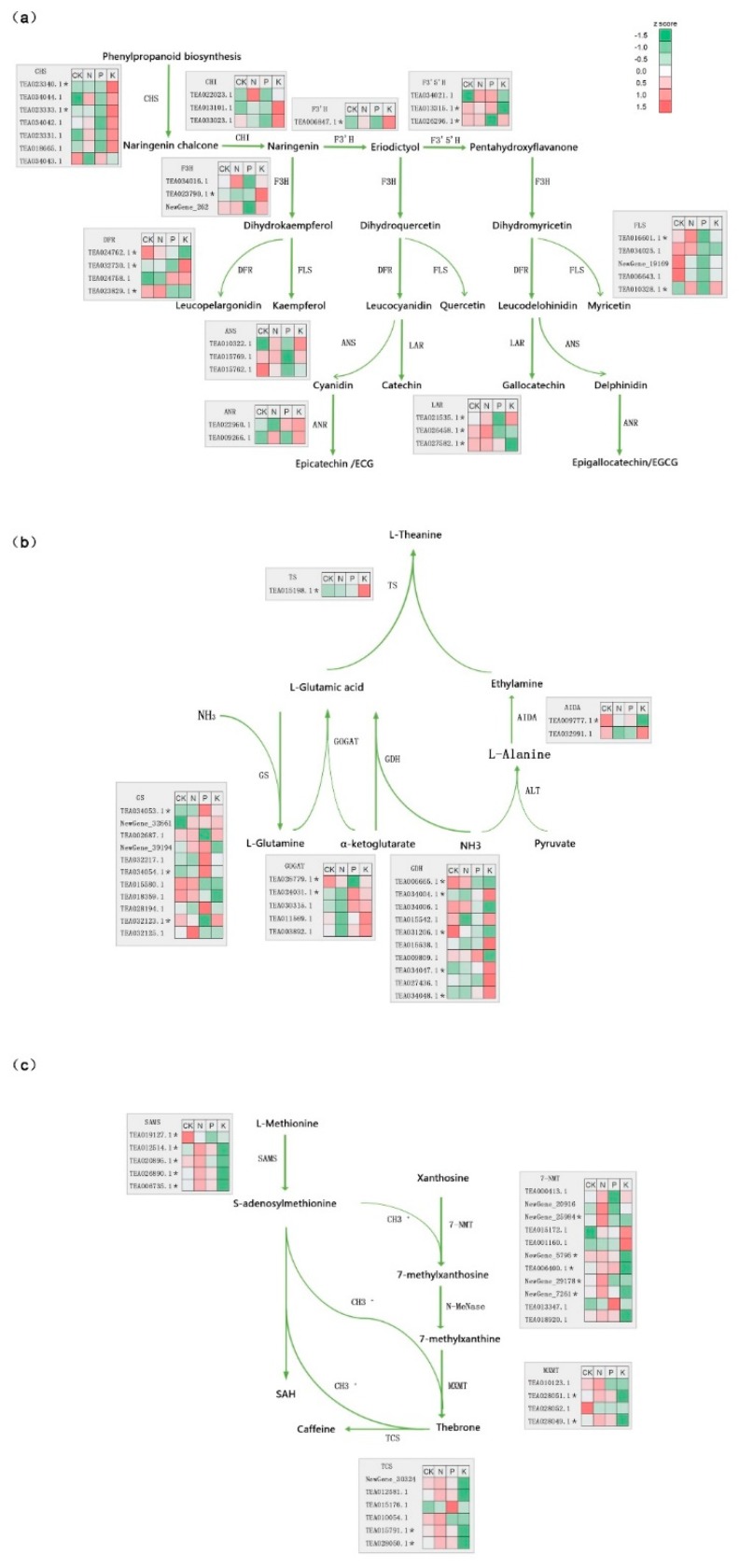
The expression of genes in biosynthesis pathway of catechins, theanine, and caffeine under N, P, and K starvation. (**a**) The expression of genes in the catechins, biosynthetic pathway. CHS, CHI, F3H, F3′H, F3′5′H, DFR, FLS, ANS, LAR, and ANR indicate genes encoding chalcone synthase, chalcone isomerase, flavanone 3-hydroxylase, flavonoid 3′-hydroxylase, flavonoid 3′,5′-hydroxylase, dihydroflavonol 4-reductase, flavonol synthase, anthocyanidin synthase, leucoanthocyanidin reductase, and anthocyanidin reductase, respectively. (**b**) The expression of genes in the l-theanine biosynthetic pathway. GS, GOGAT, GDH, TS, AIDA, and ALT represent glutamine synthetase, glutamate synthetase, glutamate dehydrogenase, l-theanine synthetase l-arginine decarboxylase, and alanine transaminase, respectively. (**c**) The expression of genes in the caffeine biosynthetic pathway. 7-NMT, MXMT, TCS, SAMS and SAH indicate xanthosine methyltransferase, theobromine synthase (7-methylxanthine methyltransferase), caffeine synthase (3,7-dimethylxanthine methyltransferase). S-adenosylmethionine synthetase, and S-adenosylhomocysteine, respectively. * refers to the differentially expressed genes among these macronutrient nutrient starvation.

Tea is rich in many secondary metabolites associated with tea quality, such as catechin derivatives (catechins), caffeine, and l-theanine [25,26,27]. Catechins that are known as flavan-3-ols are derived from phenylpropanoid and then flux into the flavonoid pathway via the catalyzation of chalcone synthase (CHS) to chalcone [28,29]. Along with the subsequent branches, ECG, EC, EGC, and EGCG, which are the main flavan-3-ols synthetized from catechin isomers, catechins are produced through a suite of crucial enzymes, including: Chalcone isomerase (CHI), flavanone 3-hydroxylase (F3H), flavonoid 3′-hydroxylase (F3′H), flavonoid 3′, 5′-hydroxylase (F3′5′H), dihydroflavonol 4-reductase (DFR), leucoanthocyanidin reductase (LAR), and anthocyanidin reductase (ANR) (Figure 5a). The expression patterns of these genes related to catechin synthesis exhibited changes that were both specific and common to the N, P, and K deficiency groups. More importantly, most of the genes encoding enzymes in these metabolite biosynthesis under different macronutrient starvation were differentially expressed, which is marked with an asterisk (Figure 5). For example, genes encoding *FLS* (TEA016601.1, TEA010328.1), *F3′H* (TEA006847.1), and *ANR* (TEA026458.1) were up-regulated following N starvation, while *DFR* (TEA024762.1) was reduced, when compared with the control group. *FLS* (TEA016601.1, TEA010328.1), *F3′H*, *F3′5′H* (TEA026296.1), *DFR* (TEA024762.1, TEA023829.1), and *LAR* (TEA021535.1, TEA026458.1) were also down-regulated following P starvation; however upon K starvation, the expression profiles were more complex and the number of responsive genes was highest. More than half of these genes were obviously induced, including *CHS* (TEA023340.1; TEA023331.1), *F3H* (TEA023790.1), *FLS* (TEA010328.1), *DFR* (TEA032730.1), and *LAR* (TEA021535.1); however, *F3′5′H* (TEA013315.1), *DFR* (TEA024762.1; TEA023829.1), and *LAR* (TEA026458.1; TEA027582.1) expression was reduced.

l-theanine is a characteristic component of tea and its biosynthetic pathway is divided into two branches. The first is catalysis of α-ketoglutarate and NH^4+^ to glutamate by GDH. The second is the well-known GS-GOGAT cycle, and both finally converge to the formation of l-theanine through the l-theanine synthetase (TS) catalysis of glutamate and ethylamine. l-theanine synthetase is the key enzyme in the process of l-theanine metabolism, therefore, it was interesting to note that the expression of *TS* was induced by the three treatments, of which K deficient treatment showed the greatest increase, while the others had a slight but not significant elevation (Figure 5b). Both P and K starvation stress up-regulated the expression of *GS* (glutamine synthetase), while no obvious changes were detected following N starvation. GOGAT-related DEGs were only tested in the P deficiency group. To some extent, *GDH* genes showed the same expression patterns following N, P, and K starvation. For example, TEA006665.1 and TEA031206.1 genes showed an obvious decline and increase, respectively, under P and K deficient conditions. Moreover, expression of TEA031206.1 was reduced in all conditions. TEA034047.1 and TEA034048.1 encoding GDH were up-regulated under P and K starvation.

Caffeine is synthesized mainly through three methylation steps, which are catalyzed by SAM-dependent N-methyltransferases (NMTs) and tea caffeine synthase (TCS) (Figure 5c). Compared with the control group, one and two DEGs were found under N and P scarcity, respectively, they were: *SAMS* (TEA019127.1), and *SAMS* (TEA019127.1), *7-NMT* (NewGene_29178). All differentially expressed genes in K starvation showed down-regulation, including four genes encoding SAMS. And as for *7-NMT*, the down-regulated genes were: NewGene 29584, NewGene 5795, TEA006400.1, NewGene_29178, NewGene_7261. MXMT encoded by TEA028051.1 and TEA028049.1 were down-regulated. In terms of TCS enzyme, two genes with down-regulation were TEA015791 and TEA028050.1. Taken together, these results implied the existence of common regulators of the responses to these stresses.

### 3.5. Changes in Major Secondary Metabolite Content Following N, P, and K Starvation

**Figure 6:**
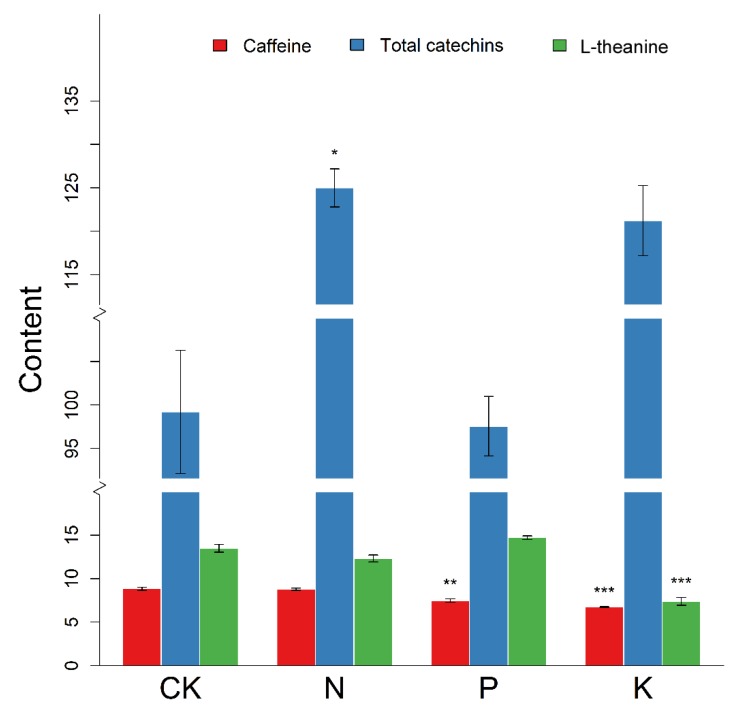
The content (mg/g) of total catechins, l-theanine, and caffeine under N, P, and K starvation. Total catechins is the sum of all individual catechin isomers, that is: Catechin(C), catechin gallate (CG), epicatechin (EC), epicatechin gallate (ECG), epigallocatechin (EGC), epigallocatechin gallate (EGCG), gallocatechin gallate (GCG), and gallocatechin (GC). Asterisks indicate the level of significance compared with the control group (CK) treated with the full nutrient solution. The error bars show the SE. Significance level is represented as stars: *, **, *** refer to *p*-values: 0.05, 0.01, and 0.001, respectively.

In order to investigate the final effect of nutrient deficiency on major secondary metabolites, we determined the content of total catechins (TC), l-theanine, and caffeine in each of the groups. As shown in Figure 6, the content of each of these metabolites differed under different nutrient starvation treatments compared with the control group. The accumulation of total catechins known as the major flavonoids was significantly increased after N starvation. As for P starvation, the reduced content of caffeine was tremendous at 1% level. Similarly, K deficiency exhibited marked inhibition in the content of caffeine. In addition, the obvious decrease in the content of l-theanine was also observed in the treatment of K limitation (*p <* 0.001). In all, these results indicated the roles of these metabolites in adaption of responsiveness to N, P, and K starvation in tea plants.

### 3.6. Analysis of Potential Regulators Involved in Metabolite Biosynthesis under N, P, and K Starvation Stress

**Figure 7:**
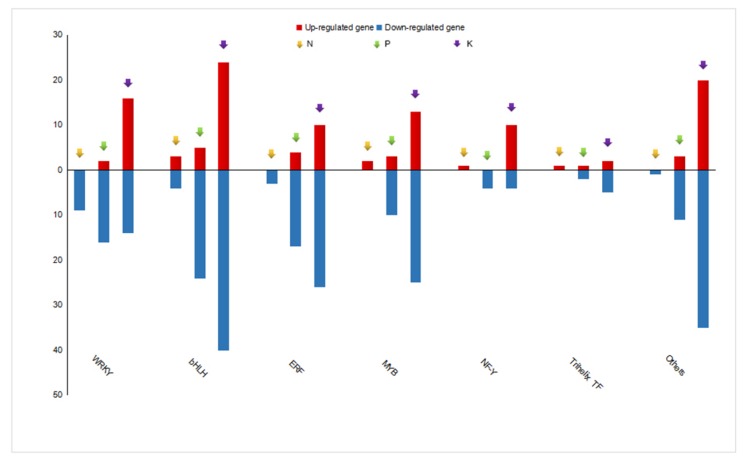
Distribution of up-regulated and down-regulated genes encoding transcription factor families in response to N, P, and K starvation. Each bar shows the number of members of the transcription factor family, with up-regulation in red and down-regulation in blue under N, P, and K starvation.

Transcription factors (TFs) play a key role in the regulation of gene expression, especially in the modulation of abiotic stress responses [30]. Numerous studies have identified members of TF families such as WRKY and MYB that act as key regulators under N, P, and K starvation [31,32,33]. We also found 278 DEGs belonging to several transcription factor families associated with N, P, and K deficiency. A total of 24, 104, and 244 responsive genes encoding TFs were identified under N, P, and K deficiency, respectively. Of those, members of the MYB, WRKY, ERF, bHLH, NF-Y, and Trihelix families were detected in each of the nutrient-starvation treatment groups (Figure 7). In the N depletion group, the highest number of up-regulated TFs was in the WRKY family (9), followed by the bHLH (7), ERF (3), MYB (2), NF-Y (1), and GARP (1), and Trihelix (1) TF families. In response to P limitation, 12 TF families were markedly changed, with bHLH (29), ERF (21), WRKY (18), and MYB (13) displaying the most abundant changes. In addition to the bHLH (64), ERF (36), MYB (38), and WRKY (30) families all contained DEGs, and some specific TF families were only represented under K depletion conditions (Tables S8–S10). We then further analyzed the expression patterns of responsive genes encoding TF family members, as shown in Figure 7. Upon the limitation of N, seven and 17 TFs with up and down-regulated expression were examined, respectively. P starvation led to 18 up-regulated and 86 down-regulated TFs. In K deficient stress, 95 and 149 TFs showed enhanced and suppressed transcription levels, respectively. Interestingly, the same expression patterns were observed for two or three TFs to different degrees. For example, five TFs that were down-regulated under all three conditions belonged to the ERF, WRKY, and bHLH families. Likewise, these TF families were also involved in N and P starved conditions with eight out of 11 TFs being down-regulated. Additionally, seven and four common TFs between the N and K deficient groups exhibited suppressed and enhanced expression levels, respectively, and belonged to the ERF, WRKY, bHLH, and Trihelix families. Furthermore, the 75 TFs were shared by the P and K limitation groups. Among these, 58 down-regulated TFs were categorized into nine TF families: ERF, WRKY, MYB, bHLH, NF-Y, E2F, HSF, NAC, GATA, and Trihelix, while nine genes encoding bHLH, WRKY, NAC, and ERF family members showed promoted expression (Table S11). Together, the similar expression patterns shown in Figure 7 indicate that the crosstalk underlying the responses to the three nutrient-deficient stresses may function through the identified TFs.

**Figure 8:**
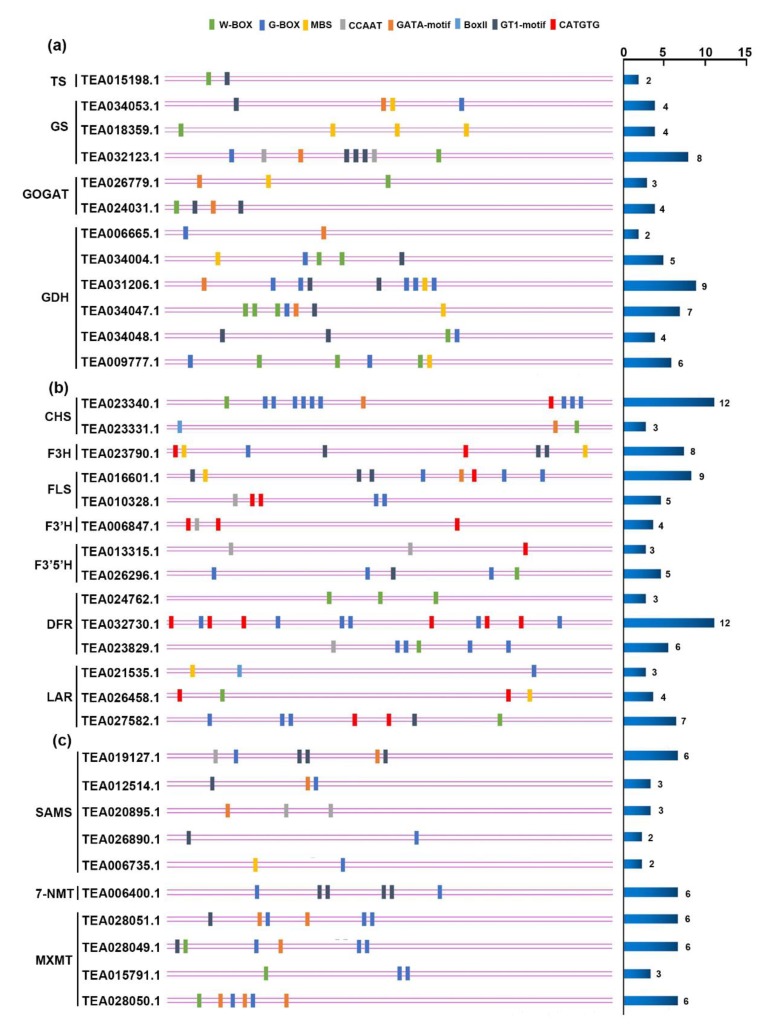
The cis-element of gene promoters in the catechin, l-theanine, and caffeine biosynthesis pathways. (**a**) The cis-element of promoters of differentially expressed genes in the catechin biosynthetic pathway. (**b**) The cis-element of promoters of differentially expressed genes in the l-theanine biosynthetic pathway. (**c**) The cis-element of promoters of differentially expressed genes in the caffeine biosynthetic pathway. The color of bar on the left represents different cis-elements bound by special transcription factor families, and the horizontal column on the right represents the total number of cis-elements of each gene.

Based on the results above and to further explore the candidate regulators of three major metabolism pathways in tea under N, P, and K starvation, we analyzed the cis-elements of promoters of DEGs from the catechin, l-theanine, and caffeine biosynthetic pathways, respectively. Since seven TF families were enriched in the response to nutrient starvation, we took them as subjects for further analysis. Interestingly, all DNA binding sites of these TFs were found in the promoter of genes encoding enzymes in metabolic pathways, except the GCC-box of ERF, suggesting that ERF, unlike others, may be modulated in an indirect manner by an ethylene signaling pathway or other network. In the catechin biosynthesis pathway, genes with the CATGTG of NAC and G-box of bHLH existed in all enzymes except F3′H. Furthermore, the W-box of WRKY was seen in the cis-element of genes encoding CHS, F3′5′H, DFR, and LAR. Moreover, genes encoding the enzymes F3H, FLS, and LAR included the MBS of MYB. Four genes, encoding FLS, F3′H, F3′5′H, and DFR, were found to have the CCAAT binding site of NF-Y. In addition, the Box II or GT1-motif of Trihelix were detected in six genes that encoded CHS, F3H, FLS, F3′5′H, and LAR, while for the GATA motif of GATA transcription factor, there were only two genes, encoding CHS and FLS. As for the genes related to l-theanine metabolic pathway, the cis-elements of their promoters were found to belong to six TF families, shown in Figure 8. It is worth noting that WRKY and Trihelix family members were identified in genes encoding all enzymes, indicating their important role in regulating l-theanine biosynthesis. Additionally, the binding sites of MYB and GATA were found in genes encoding GS, GOGAT, and GDH. However, the promoters of genes including the G-box of bHLH were only found in GS and GDH, and the CCAAT of NF-Y only in GS. Like the l-theanine pathway, there were six kinds of cis-element identified in the promoters of genes associated with the caffeine pathway. Enzymes such as SAMS, 7-NMT, and TCS contained bHLH and Trihelix binding sites. Genes encoding SAMS also contained MYB, NF-Y, and GATA binding sites, while genes encoding TCS, also had W-box and GATA-motif binding sites. Overall, the binding sites of seven kinds of responsive TFs were found in almost all major metabolite pathways, though these differed in the TF family they belonged to, which strongly demonstrates that these TFs are likely to not only connect the three nutrient starvation responses, but also to function as mediators regulating metabolite biosynthesis under N, P, and K starvation stress.

## 4. Discussion

### 4.1. Global Gene Transcriptional Changes under N, P, and K Starvation 

An increasing volume of molecular research has been conducted on tea since its beneficial and medicinal values were highlighted [17,34]. Here, we performed RNA-seq of tea to decipher the molecular responsive framework under N, P, and K starvation stress. In total, 29,431 genes were annotated and 9103 were found to differentially express under N, P, and K starvation stress, respectively, indicating nutrient-starvation stress had a great impact on tea [35,36,37]. The greatest number of differential expressed genes (DEGs) was found in the K deprivation group, with 8227, followed by P (2902) and N (781), which was consist with the results from *Arabidopsis* that the effect of K deprivation stress was more significant than starvation of other nutrients [38]. Function enrichment of DEG using GO and KEGG analysis showed the majority of DEGs were common to all nutrient deprivation conditions, indicating the close connection among nutrient starvation induced responses. GO term analysis displayed that DEGs under N starvation stress were mainly overrepresented in metabolic process, biological regulation, cell part, catalytic activity, binding, and transporter activity categories, which were also observed in P and K deficiency treatment, and similar results have been described previously for *Arabidopsis* and rice [39,40,41]. KEGG enrichment further detected many common changed pathways when treated with N, P, and K starvation, including phenylpropanoid biosynthesis, starch and sucrose metabolism, sugar metabolism, and plant hormone signal transduction pathways, suggesting that these pathways are crucial as mediators of responses to N, P, and K starvation in tea, supporting the idea that these pathways are relatively conserved in plants [40,42,43]. In all, function enrichment analysis provided basic molecular evidence for the possible crosstalk among N, P, and K starvation stress responses (Figure 2 and Figure 3), implying an intimate association between them.

NRT, a nitrate transporter is known as the core regulator of the nitrate signaling pathway by sensing nitrate level and initiating the subsequent response. In this study, N and P starvation led to no significant changes in the transcription levels of NRT, which may be the result of long-term starvation or tissue-specific expression. However, four genes encoding NRT were markedly changed, with three up-regulated and one down-regulated under K starvation, suggesting a more important role for K in tea than N and P. Interestingly, genes (TEA024685.1 and TEA032584.1) encoding AMT, which are responsible for ammonium transport, were markedly up-regulated in N and P deficiency, but were down-regulated under K deficient stress, which was reasonable since tea has a preference for ammonium as a nitrogen resource. In the phosphate signaling pathway, it has been demonstrated PSR or PHR are involved in regulating downstream phosphate starvation-induced genes [32,44,45,46]. However, PHR or its homologs PHR1-like showed obvious changes only in K starvation but not in N and P deficiency, which was supported by the fact that transcription levels were also only little affected in *Arabidopsis* and rice [44,47]. SPX-containing proteins are another key factor in phosphate starvation responses and regulate PHR at the protein level [48]. Of 12 genes encoding SPX-containing proteins, N starvation, similar to the phenomenon described above, exhibited no profound alteration, while P and K starvation changed two (both up-regulated) and four (three up-regulated and one down-regulated) genes, respectively. Additionally, the phosphate transporters, PHO1 and PHT1 are also involved in the phosphate signaling pathway [49]. Seven genes encoding PHO1 or PHT1 were found to be manipulated with three increased and four decreased expression when treated with K deficiency, whereas there were only one PHO1 up-regulated under P treatment but no significant changes following N starvation, possibly because these above-mentioned genes are mainly expressed in roots, and roots and shoots have been shown to be more responsive than leaves under N and P limitation [46]. It has been reported that K transporters and channels are essential for the responses to K starvation [50]. However, in plants treated with N starvation, only two K transporters and one K channel were modulated significantly, while genes encoding K transporters (10) and K channels (19) were found to be significantly altered following P and K starvation stress (Table S12). It was assumed that the influence of P and K starvation stress were more drastic compared to N starvation in tea. More importantly, these vital actors in the response to N, P, and K starvation highlight the existence of crosstalk among these responses through inter-mediators such as transcription factors, which are evident by an increasing number of research findings in model plants.

### 4.2. N, P, and K Starvation Differently Affect Three Major Secondary Metabolites in Tea

Recent research has focused on tea due to its unique flavor and various health benefits, which result from abundant secondary metabolites, with catechins, l-theanine, and caffeine as star components [51]. Despite advances in the identification of structural genes and TFs in secondary metabolic biosynthesis, there is still little known about responses to stress at the transcriptional level and identification of regulators under nutrient limitation stress. As shown in Figure 5, 14, 13, and 10 DEGs involved in catechins, l-theanine, and caffeine synthesis were detected under N, P, and K deficiency stress, respectively. On the whole, the expression patterns of these genes exhibited changes both specific and common to the different conditions. Strikingly, upon starvation of K, the expression profile was more complicated and the number of altered genes was greatest, indicating the response level to K was higher than others in terms of catechin biosynthesis pathways. Under N starvation, only two genes encoding GDH and AIDA were down-regulated, suggesting that the l-theanine pathway was not sensitive to N deficient stress. However, K deficiency brought about the most severe changes in expression levels of genes encoding TS, GS, GDH, and AIDA, followed by P deficiency, which affected GS, GOGAT, and GDH genes. Notably, all DEGs related to the caffeine biosynthetic pathway were down-regulated under all three nutrient limitation conditions, but the effect of K limited treatment was still the most significant, while N and P limitation down-regulated only one and two genes, respectively. In summary, N starvation had the least influence on these pathways, but K had the largest impact with the highest number of changes. In addition, accumulation differences of these metabolites further indicated the influence of N, P, and K starvation on tea, which were almost in agreement with transcriptional adaption of them. Here we provide a novel insight into response profiling and molecular bases of these three nutrient starvation conditions for further mechanism analysis.

### 4.3. Differential Expressed Transcription Factors May Regulate Metabolite Biosynthesis under N, P, and K Starvation Stress

Transcription factors (TFs) are well-known to be crucial for the regulation of gene expression at the transcriptional level and are responsive to environmental cues by binding to corresponding cis-elements of target genes [52]. In our research, DEGs encoding transcription factors mainly belonging to 14 families were identified, and almost all of them had been previously determined in transcriptional analysis in many plant species [53,54,55]. Among these, ERF, WRKY, bHLH, MYB, NF-Y, and Trihelix families were responsive under N, P, and K starvation conditions. ERF proteins were originally isolated as transcription factors that bind to the promoter regions of stress-responsive genes and are induced by various biotic and abiotic stresses [56]. For example, the suppression of AtERF070 in *Arabidopsis* led to augmented lateral root development, resulting in higher Pi accumulation [57]. RAP2.11, which belongs to the ERF family, regulates AtHAK5 expression and responds to low-potassium conditions through the regulation of other genes in the low-K signaling cascade [58]. In addition, several members of the MYB TF family induced by N depletion in *Arabidopsis* were isolated [59,60]. A novel MYB coiled-coil type transcription factor, OsMYBc in rice, was found to bind to the *OsHKT1;1* promoter, indicating its potential function in K deficiency [61]. The bHLH family is prevalent in the adaption to nutrient stress. For example, bHLH32 in *Arabidopsis* and OsPTF1 in rice were reported to respond to Pi starvation [62]. In response to nitrogen depletion, GL3, which belongs to the bHLH TF family, is strongly induced, and anthocyanin synthesis is activated in seedlings and rosette stage plants [63]. To our knowledge, several members of WRKY families have been verified to respond to phosphate starvation in *Arabidopsis*, rice, and wheat [64,65,66,67,68]. The WRKY family in tea is characterized only in structure but not in biological function [69]. NF-Y and Trihelix members are also associated with abiotic stress but were not functionally characterized in the present study [70]. There were another one, six, and seven TF families that were also associated with N, P, and K starvation, respectively. Since little is currently known about the biological roles of genes involved in macronutrient limitation in tea, our results highlight that these TFs may play crucial roles in response to N, Pm and K starvation and can be used for further functional analysis as candidate regulators.

To explore whether the identified TFs have a regulatory function on genes in three major metabolite biosynthesis pathways, we analyzed the cis-element promoter of structural genes based on the eight TF families with the highest number of overrepresented genes. To our delight, the binding sites of almost all TFs were identified in the promoter regions of genes, with the exception of the GCC box of ERF, which was not detected in any genes from these pathways, indicating that the ERF response to nutrient starvation has an indirect role in the regulation of these metabolism pathways. 

By integrating transcriptomic and metabolic profiling data, 35 TFs were identified to be significantly correlated with total catechin changes, including MYB, WRKY, bHLH, and ERF, suggesting these TFs may both act as regulatory and responsive genes for the three macronutrients studied [29]. A TF regulation network performed in tea revealed that bZIP, bHLH, and MYB families are associated with caffeine biosynthesis, whereas WRKY is related to the l-theanine pathway [49]. Furthermore, several TF families previously characterized to be involved in abiotic stresses were also detected in this study, such as WRKY and bHLH [69,71]. When fed with different N forms, transcription analysis in tea showed that MYB, bHLH, WRKY are likely correlated to major secondary metabolites, while several TFs such as GATA and NAC were not found, probably due to differences in the manner of N treatment. Taken together, both structural and regulatory genes in the catechin, l-theanine, and caffeine biosynthesis pathways were regulated by more than one TF family. Up to now, only a few TFs belonging to the MYB, bHLH, WD40, and WRKY families have been characterized functionally in tea [72,73,74,75]. Hence, based on our findings, it is hypothesized that those identified TFs are not only responsive to nutrient starvation but also may act as regulators of three major metabolites in tea. 

## 5. Conclusions

In summary, since tea is often subjected to N, P, and K deprivation stress, which influences its quality and medical value, we carried out RNA-seq of tea under those macronutrient limitation conditions to explore the genome-wide response mechanisms. Here, we identified 9103 differential expressed genes (DEGs) in total by RNA-seq. In addition, function enrichment analysis using GO and KEGG suggested the potential molecular interaction between each of the genes. Furthermore, the effect of N, P, and K starvation on three major metabolism pathways revealed that responses were not specific to the condition, but changes in gene expression were similar. We also found genes encoding members of 14 transcription factor families that were differentially expressed under N, P, and K deficiency. Furthermore, the cis-element analysis of gene promoters uncovered that members of WRKY, MYB, bHLH, NF-Y, NAC, Trihelix, and GATA families were more likely to regulate genes involved in the three major metabolite pathways. Taken together, these findings indicate that N, P, and K starvation have an impact on catechins, l-theanine, and caffeine biosynthetic pathways and have potential crosstalk among each other through shared responsive transcription factors and other inter-mediators. In all, our results provide a comprehensive insight to allow further studies on mechanisms of responses to these macronutrient stresses.

## Supplementary Materials

The following are available online at https://www.mdpi.com/2073-4425/11/3/241/s1, Table S1: The components and concentration of N, P, K starvation and full nutrition solution, Table S2: The relative expression of responsive genes involved in N, P, and K starvation, Table S3: The expression of all identified genes under N, P, and K starvation, Table S4: Annotation of all identified genes under N, P, and K starvation, Table S5: The expression of differentially expressed genes under N starvation, Table S6: The expression of differentially expressed genes under P starvation, Table S7: The expression of differentially expressed genes under K starvation, Table S8: Distribution of differentially expressed genes encoding TF under N starvation, Table S9: Distribution of differentially expressed genes encoding TF under P starvation, Table S10: Distribution of differentially expressed genes encoding TF under K starvation, Table S11: The regulated pattern of shared DEGs among N, P, and K starvation, Table S12: The list of differentially expressed genes encoding key members involved in response to N, P, and K starvation.
